# Bioinformatics Analyses of the Transcriptome Reveal Ube3a-Dependent Effects on Mitochondrial-Related Pathways

**DOI:** 10.3390/ijms21114156

**Published:** 2020-06-10

**Authors:** Julia Panov, Lilach Simchi, Yonatan Feuermann, Hanoch Kaphzan

**Affiliations:** Sagol Department of Neurobiology, Faculty of Natural Sciences, University of Haifa, Abba Khoushy Ave 199, Haifa 3498838, Israel; juliapanov.uni@gmail.com (J.P.); simchi.lilach@gmail.com (L.S.); yfeuerman@univ.haifa.ac.il (Y.F.)

**Keywords:** Angelman syndrome, mitochondria, oxidative stress, reactive oxygen species, gene expression, machine learning, bioinformatics

## Abstract

The *UBE3A* gene encodes the ubiquitin E3-ligase protein, UBE3A, which is implicated in severe neurodevelopmental disorders. Lack of UBE3A expression results in Angelman syndrome, while UBE3A overexpression, due to genomic 15q duplication, results in autism. The cellular roles of UBE3A are not fully understood, yet a growing body of evidence indicates that these disorders involve mitochondrial dysfunction and increased oxidative stress. We utilized bioinformatics approaches to delineate the effects of murine *Ube3a* deletion on the expression of mitochondrial-related genes and pathways. For this, we generated an mRNA sequencing dataset from mouse embryonic fibroblasts (MEFs) in which both alleles of *Ube3a* gene were deleted and their wild-type controls. Since oxidative stress and mitochondrial dysregulation might not be exhibited in the resting baseline state, we also activated mitochondrial functioning in the cells of these two genotypes using TNFα application. Transcriptomes of the four groups of MEFs, *Ube3a*^+/+^ and *Ube3a*^−/−^, with or without the application of TNFα, were analyzed using various bioinformatics tools and machine learning approaches. Our results indicate that *Ube3a* deletion affects the gene expression profiles of mitochondrial-associated pathways. We further confirmed these results by analyzing other publicly available human transcriptome datasets of Angelman syndrome and 15q duplication syndrome.

## 1. Introduction

The *UBE3A* gene that encodes for the ubiquitin E3-ligase protein UBE3A is located in the q11–q13 region of chromosome 15 in humans, and at 28.65 Cm of chromosome 7 in mice. UBE3A possesses five well-characterized functional domains: a HECT domain, E6 binding domain, p53 binding domain, three nuclear receptor interaction domains, and an activation domain [1,2]. To date, UBE3A expression has been observed to be ubiquitous in many tissues [3,4]. So far, it was shown that UBE3A has two main functions: as a hormone-dependent coactivator of nuclear hormone receptors, such as androgen and estrogen receptors [5], and as an E3 ligase of the HECT domain family, which catalyzes ubiquitin transfer to the substrate protein, leading to its degradation [6]. These two functions of nuclear hormone receptor coactivator and ubiquitin–protein ligase activity were shown to be independent [1,7,8]. To date, three main Ube3a isoforms have been identified in the mouse and shown to be active [9]. Recent evidence suggests that one of the isoforms is localized to the nucleus, suggesting additional potential roles of Ube3a [10,11,12]. In spite of numerous studies regarding UBE3A, its cellular roles are not fully understood. However, it has been implicated in regulating processes that are associated with mitochondrial functioning [13,14,15], such as cell proliferation and apoptosis [8,16,17,18,19]. Furthermore, several studies showed a more direct link between aberrant mitochondrial functioning and altered expression levels of the *UBE3A* gene. While the cellular roles of UBE3A are not fully understood, it is known to play a role in neurodevelopmental disorders in a dose-dependent manner. Lack of UBE3A causes a severe neurodevelopmental disorder known as Angelman syndrome (AS) [20], while overexpression of UBE3A, as in the case of 15q duplication (dup15q), leads to severe autism [21,22,23,24,25]. In addition to UBE3A roles in neurodevelopment, alterations in UBE3A levels were associated with cancers such as cervical cancer, prostate cancer, and breast cancer [8,17,18,26,27,28,29]. 

One of the first studies that examined the cellular functions of *UBE3A* showed that in dup15q autism, where UBE3A is overexpressed, there is a mitochondrial dysfunction [30]. Several other studies that investigated the lack of UBE3A expression also showed mitochondrial dysfunction. The first of these studies showed that lack of UBE3A expression in the hippocampus of AS model mice correlates with mitochondrial dysfunction and impaired morphology of the inner mitochondrial membrane [31]. This study also demonstrated reduced complex-III oxidative phosphorylation in whole-brain mitochondria of AS model mice [31]. Later on, it was shown that AS mice have increased oxidative stress in the hippocampus. Treating mitochondrial-generated oxidative stress with mitoQ, a mitochdria-targeted antioxidant [32], was able to reduce this oxidative stress and rescued several hippocampal-dependent deficits [33]. Coinciding with these results, another study showed that administration of a CoQ10 analogue, idebenone, to AS model mice improved the expression of complexes III and IV in the hippocampus and in the cerebellum and also corrected motor coordination and anxiety levels [34]. The findings of enhanced oxidative stress were also replicated with in vivo imaging with QUEST-MRI in a similar AS model mouse [35]. A later study that used electron microscopy found association of UBE3A molecules with the outer membrane of the mitochondria [36]. Taken together, these findings suggest that the absence of UBE3A alters the expression profiles of mitochondria-related pathways and processes. 

It is possible that in the resting baseline conditions, impaired mitochondrial functioning might suffice and would not reflect altered signaling pathways. However, further challenging the mitochondria will expose additional compromised mitochondria-related pathways. One way to induce mitochondrial-related signaling pathways is to stimulate cells with the cytokine tumor necrosis factor-α (TNFα). TNFα stimulation has been shown to elicit a wide range of cellular responses in almost all cell types, including cell death, survival, differentiation, and proliferation [37,38,39], and many of these activated pathways involve or affect the mitochondria. For example, TNFα stimulation induced translocation of mitochondria from a dispersed distribution to a perinuclear cluster [39]. This TNFα-induced mitochondrial translocation and clustering is a step in the induction of apoptosis by TNFα [39]. In addition, TNFα induces the formation of reactive oxygen species (ROS) in the mitochondria [40,41,42] by activating the mitochondrial electron transport chain [41]. This was shown in several cell types as well as in isolated mitochondria [43,44]. Beyond the acceleration of ROS production, in intestinal epithelial cells and in HT-22 neuronal-derived cells, TNFα also induced mitochondrial dysfunction, which resulted in a reduction of the mitochondrial membrane potential and in a decreased oxygen consumption [40,42]. 

In addition, mitochondrial dysfunction [45,46,47,48,49,50,51] as well as TNFα signaling [52,53] were implicated in various neurodevelopmental disorders [54]. Hence, understanding the effects of *Ube3a* deletion on mitochondrial-related signaling pathways might shed light on the development of autism spectrum disorders beyond AS and dup15q. 

Given all of the above, our aim in this study was to investigate the effects of *Ube3a* deletion on the expression of mitochondrial-related genes, by also utilizing TNFα stimulation as a means to further push mitochondrial-related pathways. For this, we utilized mouse embryonic fibroblasts (MEFs) that either contained both copies of the *Ube3a* gene (*Ube3a*^+/+^) or a complete deletion of the two copies of *Ube3a* gene (*Ube3a*^−/−^). Within these cell cultures, we examined the mRNA expression profiles of genes that encode for proteins directly localized to the mitochondria [55] and of genes that are part of mitochondrial-related pathways. In addition, in order to validate the abovementioned results in other AS models, namely, how *Ube3a* deletion effects the expression of genes localized to mitochondria, we utilized additional previously published RNA-sequencing and proteomics datasets. The first transcriptome dataset, used for this validation, is an RNA-sequencing dataset we recently generated from brains (ventral hippocampal region) of AS model mice and their wild-type (WT) control littermates [56]. The second publicly available transcriptome dataset we used for validation was generated from iPSC-derived neurons from AS patients and healthy donor controls [57]. Moreover, we further compared our results to previously reported proteomics changes found in three different brain regions (cerebellum, cortex, and hippocampus) in AS model mice compared to their WT controls [58]. Furthermore, we contrasted our transcriptome analyses of human iPSC-derived neurons from AS patients to the previously published transcriptome changes found in postmortem brains of dup15q autism patients [59]. 

## 2. Results

### 2.1. Ube3a Deletion Affects the Expression of Genes Involved in Mitochondrial Functioning 

**Figure 1:**
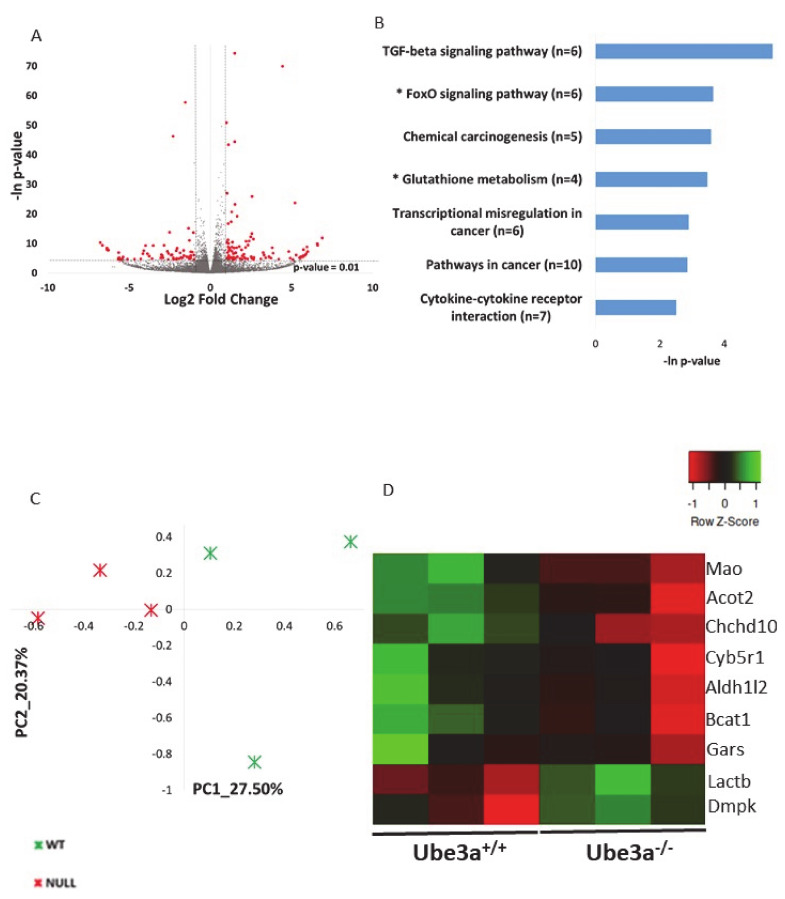
*Ube3a* deletion alters the expression of genes, thus affecting mitochondrial-related pathways in the resting baseline condition. **A.** Volcano plot showing the distribution of the gene expression fold changes (x-axis) and *p*-values (y-axis) in *Ube3a*^+/+^ and *Ube3a*^−/−^ MEFs. Genes with absolute fold change > 2 and *p*-value < 0.01 are indicated in red. **B.** Functional pathway enrichment analysis of differentially expressed genes in *Ube3a*^+/+^ compared to *Ube3a*^−/−^ MEFs. The y-axis shows significantly enriched pathways (FDR < 0.05). The x-axis indicates *p-*value of enrichment of the given pathway. Asterisk (*) denotes pathways associated with mitochondrial functioning or with production of reactive oxygen species. **C.** Principal component analysis (PCA) based on expression of genes localized to mitochondria (MitoCarta2) in *Ube3a*^+/+^ and *Ube3a*^−/−^ MEFs. The PCA reveals a clear separation of *Ube3a*^+/+^ (green marks) and *Ube3a*^−/−^ (red marks) along the main PC1-PC2 components. **D.** Heat map showing expression patterns of nine differentially expressed genes coding for proteins localized to mitochondria in *Ube3a*^+/+^ and *Ube3a*^−/−^ MEFs. The heat map indicates upregulation (green), downregulation (red), and unaltered gene expression (black). The columns represent individual samples.

As aforementioned, a growing body of evidence shows that *Ube3a* deletion is related to mitochondrial dysfunction and dysregulation of mitochondrial-dependent oxidative stress, but the cause for that is not known. It is a possible that these mitochondrial-related impairments are due to perturbation of gene expression regulation. Nevertheless, no studies examined the effects of *Ube3a* deletion on the expression of mitochondrial-related genes. Using transcriptome sequencing and applying various bioinformatics tools and approaches gives the opportunity to examine such questions in a broad perspective of pathways and cellular processes, instead of looking into particular genes. To examine whether indeed there is an indication for the involvement of mitochondrial-related transcriptome, we generated poly-A RNA sequencing of *Ube3a*^+/+^ and *Ube3a*^−/−^ mouse embryonic fibroblasts (MEFs) derived from the same litters. Next, we performed a broad unsupervised analysis to identify differentially expressed genes in these *Ube3a*^−/−^ cells compared to *Ube3a*^+/+^ cells by utilizing DeSeq2 algorithm [60]. We found that 121 genes were upregulated and 65 were downregulated in *Ube3a*^−/−^ cells compared to *Ube3a*^+/+^ cells (Figure 1A, Table S1). Functional enrichment analysis revealed several pathways dysregulated in *Ube3a*^−/−^ MEFs (Figure 1B). Several of the enriched pathways are directly related to mitochondrial-dependent oxidative stress: glutathione pathway and FoxO pathway (Figure S1) [61,62]. Nevertheless, the other enriched pathways are also indirectly related to mitochondrial functioning. This unsupervised analysis shows a link between the *Ube3a*-dependent transcriptome and mitochondrial-dependent pathways. To further reveal if there is a link between *Ube3a* deletion and mitochondrial-related transcriptome, we utilized a MitoCarta2 database of proteins that are localized to the mitochondria, which consists of 1158 proteins [55]. We found that 1080 genes known to localize to the mitochondria are expressed in our transcriptome data set (Table S2). Performing principal component analysis (PCA) on this gene expression matrix revealed that these genes clearly differentiate between the *Ube3a*^+/+^ and *Ube3a*^−/−^ MEFs (Figure 1C). However, while most of these genes show small insignificant differences in expression, only nine genes were significantly altered in *Ube3a* knockout cells (Figure 1D, Table S3). This finding of only a small number of genes significantly altered with *Ube3a* deletion is not surprising. These cells are in a quiescent state, without any stress or mitochondria-relevant stimulation, thus both *Ube3a*^+/+^ and *Ube3a*^−/−^ cells are expected to have a relatively preserved and stable homeostasis. Hence, to reveal mitochondrial-related transcriptomic differences, it is essential to induce some perturbation that will stimulate the mitochondrial-related pathways.

### 2.2. TNFα Induces Significant Changes in Ube3a^+/+^ and Ube3a^−/−^ Cells 

To induce a mitochondrial-related perturbation, we treated the two types of cells (*Ube3a*^+/+^ and *Ube3a*^−/−^ MEFs) with TNFα. TNFα stimulation is known to affect mitochondrial functioning and the induction of mitochondrial-related stress. TNFα was shown to induce mitochondria translocation [39]. Furthermore, it was shown that TNFα induces stress leading to mitochondrial dysfunction and a drop in mitochondrial membrane potential, and stimulates mitochondrial reactive oxygen species (ROS) production [40,41,42].

**Figure 2:**
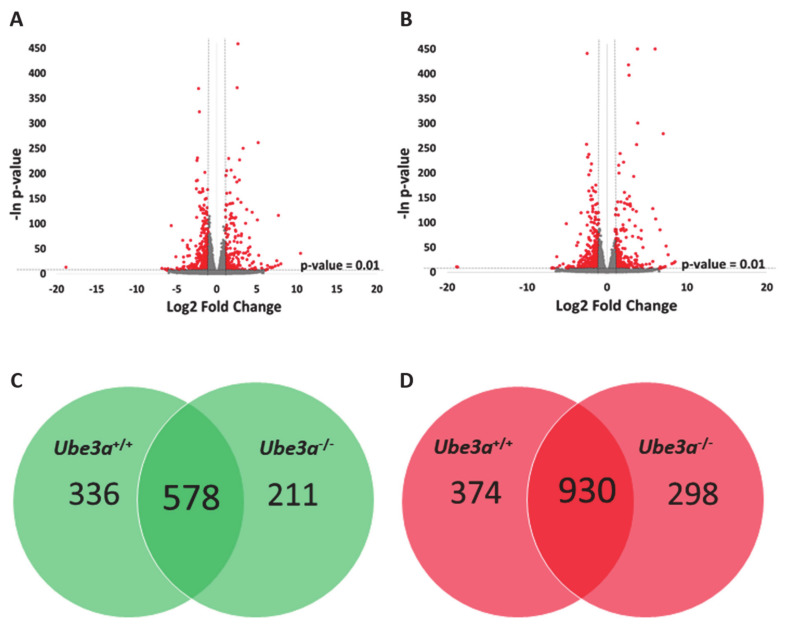
TNFα treatment alters the gene expression profiles in *Ube3a*^+/+^ and *Ube3a*^−/−^ MEFs. **A.** Volcano plot showing the distribution of the genes by log2 expression fold changes induced with TNFα stimulation relative to no treatment (x-axis) and –ln of *p-*values (y-axis) in *Ube3a*^+/+^ MEFs. Genes with absolute fold change >2 and *p-*value < 0.01 are indicated in red. **B.** Volcano plot showing the distribution of the genes by log2 expression fold changes induced with TNFα stimulation relative to no treatment (x-axis) and –ln of *p-*values (y-axis) in *Ube3a*^−/−^ MEFs. Genes with absolute fold change >2 and *p-*value < 0.01 are indicated in red. **C.** Venn diagram indicating the number of significantly upregulated genes due to TNFα treatment in *Ube3a*^+/+^ and *Ube3a*^−/−^ MEFs. **D.** Venn diagram indicating the number of significantly downregulated genes due to TNFα treatment in *Ube3a*^+/+^ and *Ube3a*^−/−^ MEFs.

**Figure 3:**
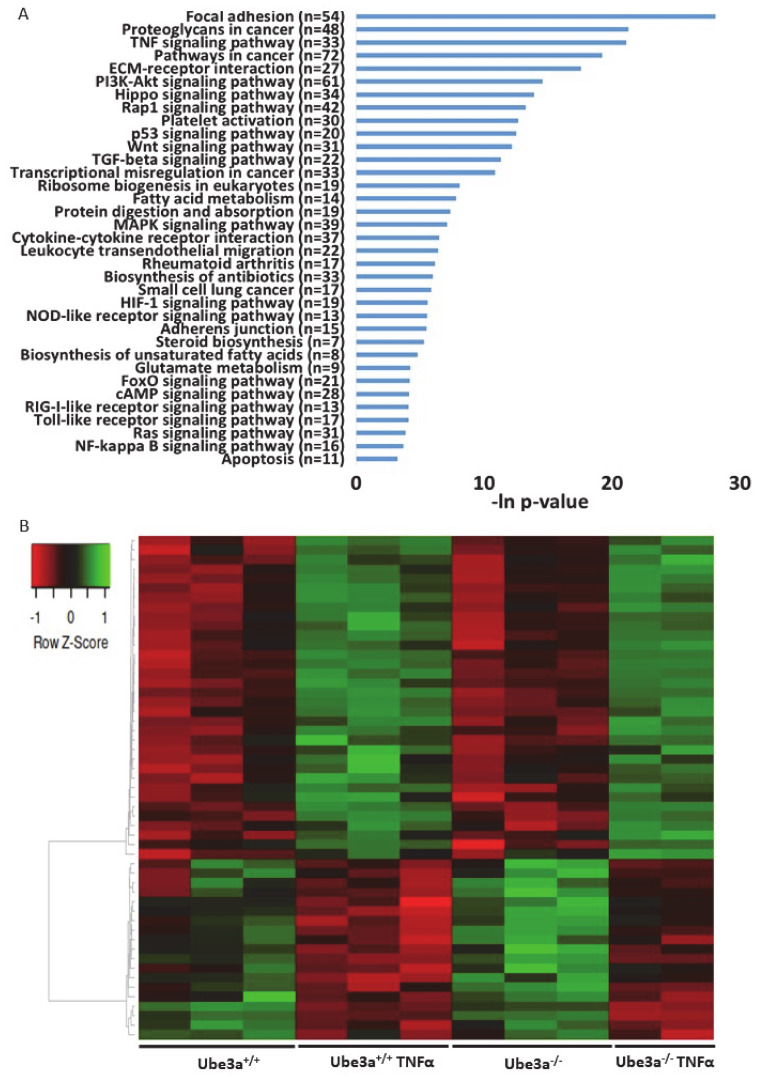
TNFα treatment alters the gene expression profiles in *Ube3a*^+/+^ and *Ube3a*^−/−^ MEFs. **A.** Functional pathway enrichment analysis of genes found to be differentially expressed in both TNFα-treated *Ube3a*^+/+^ compared to untreated *Ube3a*^+/+^ and in TNFα-treated *Ube3a*^−/−^ compared to untreated *Ube3a*^−/−^ MEFs. The y-axis shows significantly enriched pathways (FDR < 0.05). The x-axis indicates –ln *p-*value of enrichment of the pathways. **B.** Heat map showing the expression patterns of mitochondrial-localized genes in *Ube3a*^+/+^ and *Ube3a*^−/−^ MEFs with and without TNFα treatment. Green indicates upregulation, red indicates downregulation, and black indicates no change in gene expression. The columns represent individual samples.

To assess the differences of gene expression profiles in response to TNFα stimulation, we treated half of the samples of *Ube3a*^+/+^ and *Ube3a*^−/−^ MEFs with TNFα and half of the samples were untreated. We harvested the cells 16 h later and generated an mRNA transcriptome of these four groups. As expected, TNFα stimulation induced significant changes in gene expression profiles in both *Ube3a*^+/+^ and *Ube3a*^−/−^ cells, when compared to untreated cells (Figure 2A–B, Tables S4 and S5). Differential gene expression analysis revealed 914 genes that were significantly upregulated and 1304 genes that were significantly downregulated in *Ube3a*^+/+^ following treatment with TNFα compared to untreated *Ube3a*^+/+^ MEFs. For the *Ube3a*^−/−^ cells, we identified 789 upregulated and 1228 downregulated genes in TNFα-treated *Ube3a*^−/−^ compared to untreated *Ube3a*^−/−^ MEFs. Common to both *Ube3a*^+/+^ and *Ube3a*^−/−^ cells, 578 genes were significantly upregulated and 930 genes were significantly downregulated when treated with TNFα (Figure 2C–D, Table S6). Overall, 1508 genes showed significant response to TNFα in both *Ube3a*^+/+^ and *Ube3a*^−/−^ MEFs. Enrichment pathway analysis of these 1508 genes showed some pathways that are directly linked to mitochondrial functioning (fatty acid metabolism [63], aspartate and glutamate metabolism [64], and steroid biosynthesis [65]), or to mitochondrial-related oxidative stress (HIF1 signaling [66] and FoxO signaling [61]) (Figure 3A). Of these 1508 genes, 53 genes were mitochondrial-localized genes (Figure 3B, Table S7). 

### 2.3. TNFα Differentially Affects Mitochondria-Associated Pathways in Ube3a Knockout MEFs

**Figure 4:**
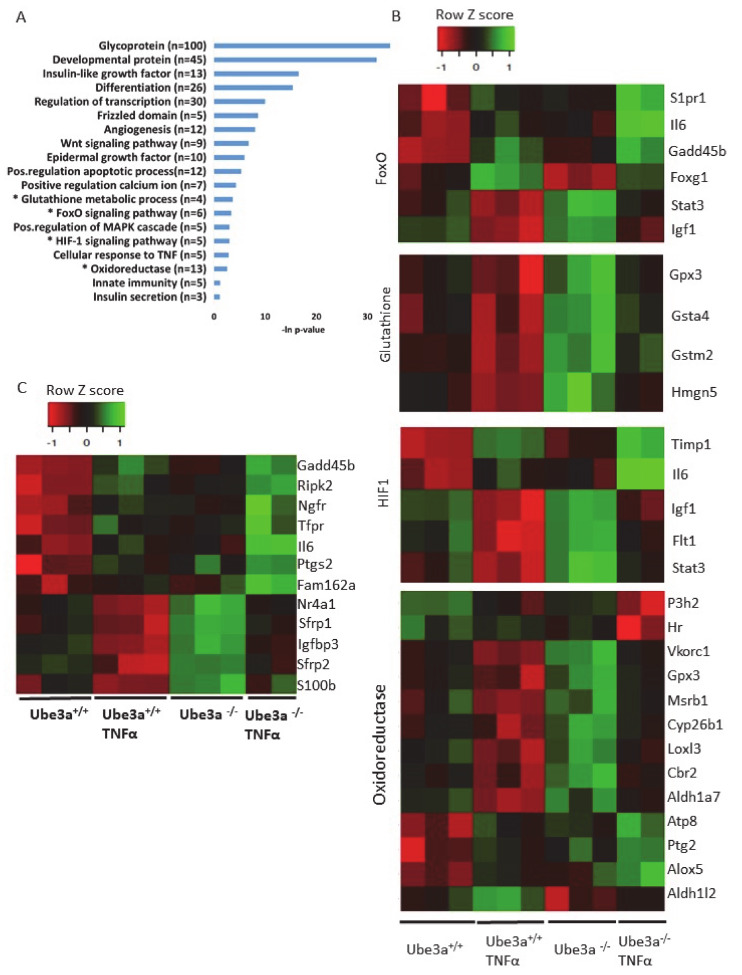
Differentially affected genes in *Ube3a*^+/+^ and *Ube3a*^−/−^ MEFs due to TNFα stimulation. Factor regression analysis for the two factors of genotype and TNFα treatment (2×2) identifies differentially affected genes and their related pathways. **A.** Functional pathway enrichment analysis of 275 significant differentially affected genes. Significant genes were extracted by factor regression analysis of two factors: genotype and TNFα treatment (Benjamini–Hochberg adjusted *p-*value < 0.01). The y-axis shows significantly enriched pathways (FDR < 0.05). The x-axis indicates –ln *p-*value of enrichment of the given pathways. Asterisk (*) denotes processes directly associated with mitochondrial functioning or with production of reactive oxygen species. **B.** Heat map showing the expression patterns of differentially affected genes (by two factors, genotype and TNFα treatment) that are included in biological processes that are directly associated with mitochondrial functioning (glutathione metabolic pathway, FoxO pathway, HIF-1 pathway, and oxidoreductase process). In the heat map, green indicates upregulation, red downregulation, and black unchanged gene expression. The columns represent individual samples. **C.** Heat map showing the expression patterns of differentially affected genes (by two factors, genotype and TNFα treatment) that are included in the pathway of positive regulation of apoptotic processes. In the heat map, green indicates upregulation, red downregulation, and black unchanged gene expression. The columns represent individual samples.

**Figure 5:**
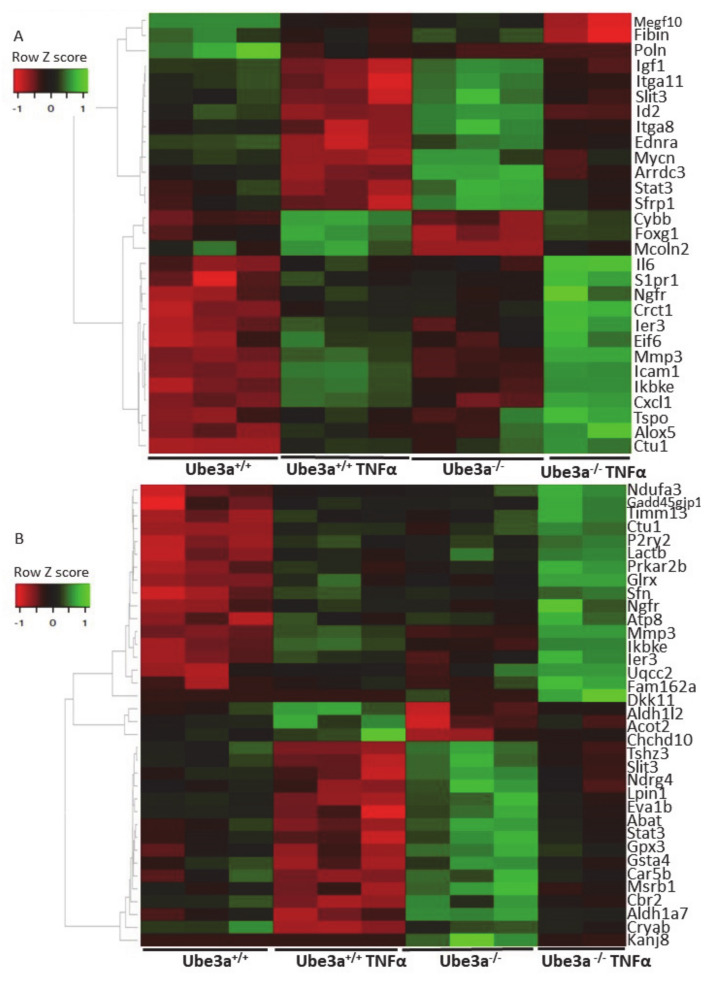
The expression profiles of mitochondrial-related genes are differentially affected by TNFα stimulation in *Ube3a*^+/+^ and *Ube3a*^−/−^ MEFs. **A.** Heat map showing the expression patterns of differentially affected genes that are associated with the ROS metabolic processes pathway. **B.** Heat map showing the expression patterns of differentially affected genes that are localized to mitochondria. Significant genes were extracted by factor regression analysis for the two factors of genotype and TNFα treatment (Benjamini–Hochberg adjusted *p-*value < 0.01). Green indicates upregulation, red indicates downregulation, and black indicates no change in expression. The columns represent individual samples.

The previous analyses demonstrated some differences in the expression profiles of mitochondrial-related genes, but our prediction was that application of mitochondria-relevant stimulation might further emphasize such differences. As can be seen in the previous analysis, 547 genes showed significant upregulation in only one of the genotypes (Figure 2C) and 672 genes showed significant downregulation in only one of the two genotypes. Nevertheless, this analysis does not mean that the differential responses to TNFα stimulation, meaning the differences in the change of the expression of these genes, were significant between the two genotypes. A PCA of the expression profiles matrix of all genes showed that the four groups of genotype and treatment were considerably different (Supplementary Figure S2). To further delineate the differences in the induction of gene expression profiles by TNFα stimulation between *Ube3a*^+/+^ and *Ube3a*^−/−^ MEFs, we performed 2 × 2 factor regression analysis (see Methods) considering the two factors of genotype and TNFα treatment. This analysis identified 275 genes significantly affected by both factors: genotype and TNFα treatment (Benjamini–Hochberg adjusted for multiple comparisons *p* < 0.01) (Table S8). Pathway enrichment analysis of these 275 genes revealed many ROS-associated pathways, such as glutathione metabolic process, FoxO signaling pathway, HIF-1 signaling pathway, and oxidoreductase pathway (Figure 4A–B, Table S9). All these pathways are tightly associated with ROS production due to mitochondrial dysfunction [61,62,66,67,68], as well as the positive regulation of apoptosis pathway that included 12 differentially affected genes that are all associated with regulating mitochondrial function (Figure 4C, Table S10). To follow through this analysis, we also examined the genes within the pathway termed as ‘ROS metabolic process’ and found 29 genes that were differentially affected by genotype and TNFα treatment (Figure 5A, Table S11). Examining the mitochondrial-localized proteins from MitoCarta2 [55], we found 36 differentially affected genes by genotype and TNFα treatment (Figure 5B, Table S12). All these results together suggest that Ube3a affects the expression of mitochondria-associated genes under TNFα perturbation.

### 2.4. Additional Transcriptomics Datasets from other AS Models Show Differential Expression of Mitochondrial-Localized Genes

**Figure 6:**
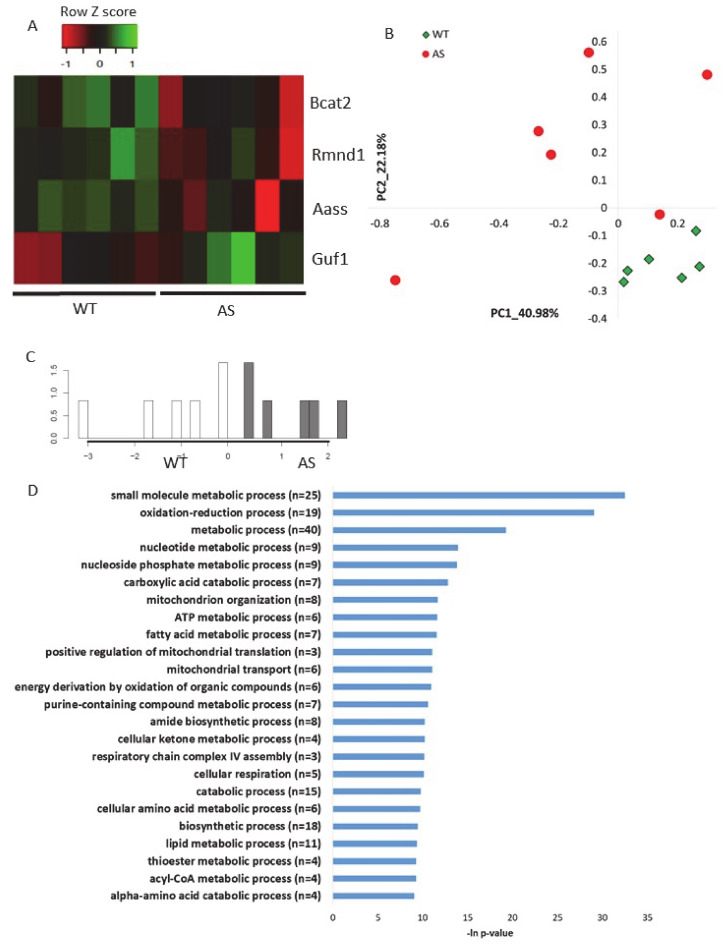
Genes that encode for mitochondrial-localized proteins are dysregulated in the hippocampal region of AS model mice **A.** Heat map showing the expression patterns of differentially expressed genes that encode for mitochondrial-localized proteins in hippocampi of AS model mice and WT littermates (*p-*value < 0.05). Green indicates upregulation, red indicates downregulation, and black indicates no change in expression. The columns represent individual samples. **B.** Principal Component Analysis (PCA) of 50 mitochondrial-localized genes expressed in mice hippocampi, which were identified by multirun RF procedure as classifiers of WT and AS genotypes. **C.** LDA histogram plot of WT and AS mice hippocampi samples based on the expression of 50 mitochondrial-localized genes that were identified by multirun RF procedure as classifiers of WT and AS genotypes. **D.** Functional pathway enrichment analysis of 50 mitochondrial-localized genes that were identified by multirun RF procedure as classifiers of WT and AS genotypes. The y-axis shows significantly enriched biological processes (FDR < 0.05). The x-axis indicates –ln *p-*value of enrichment of the given process.

To validate the abovementioned results in other AS models, namely, how *Ube3a* deletion affects the expression of genes localized to mitochondria, we utilized two additional previously published RNA-sequencing datasets. The first dataset was a transcriptomics dataset recently generated by us from brains (hippocampal region) of AS model mice and their control littermates [56] (see Section 4). This analysis showed that four mitochondrial-localized genes were differentially expressed (*p-*value < 0.05) in AS and WT mouse hippocampi (Figure 6A, Table S13). Following, we applied a multirun random forest (multirun RF) procedure (see Section 4) to identify whether mitochondrial-localized genes, as a group, can differentiate between AS and WT hippocampi. This analysis of multirun RF yielded a list of 50 classifier genes (Table S14). Based on the expression of these genes, we performed unsupervised PCA, which showed a clear separation between the two genotypes (Figure 6B). In addition, we performed a supervised linear discriminant analysis (LDA) that showed also a perfect separation with 100% predictability between the AS and the WT mouse hippocampi (Figure 6C). These clear PCA and LDA results confirmed that genes chosen by the multirun RF procedure are indeed good classifiers differentiating between AS and WT mouse hippocampi samples. Pathway enrichment analysis of these chosen genes revealed that they are involved in the various aspects of mitochondrial functioning processes: oxidation–reduction, mitochondrial organization, mitochondrial translation, mitochondrial transport, and respiratory chain complex IV assembly (Figure 6D).

**Figure 7:**
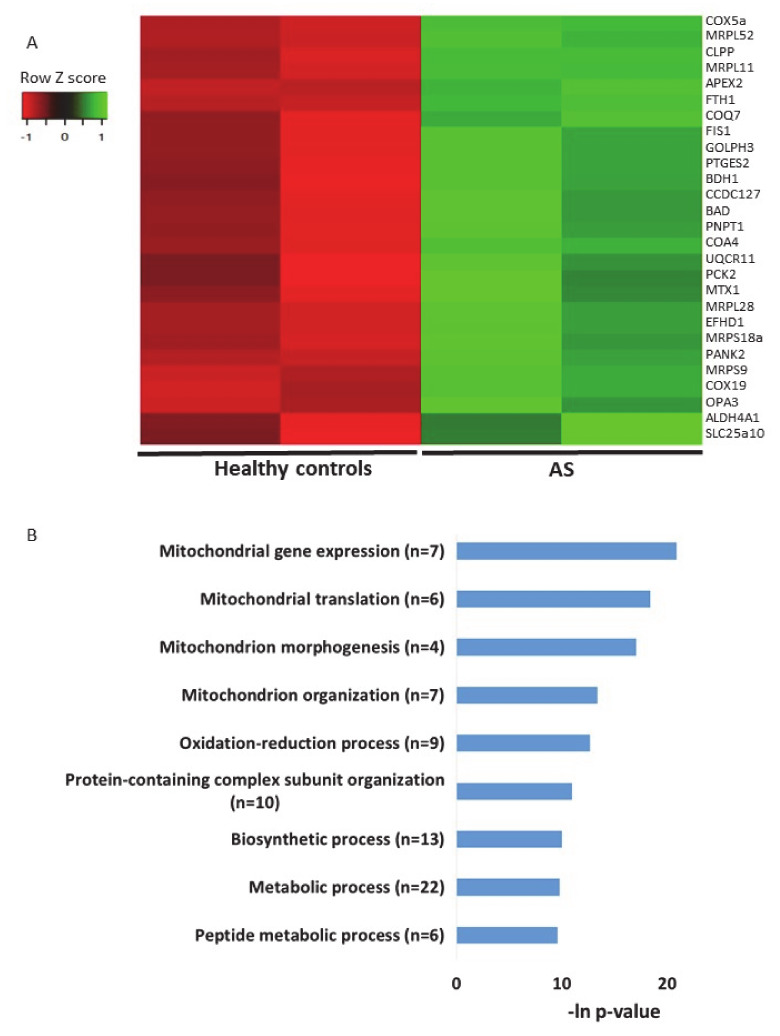
Genes of mitochondrial-localized proteins are dysregulated in iPSC-derived neurons from Angelman syndrome patients and healthy controls. **A.** Heat map showing the expression patterns of differentially expressed genes that encode for mitochondrial-localized proteins in iPSC-derived neurons taken from AS patients and healthy controls (*p-*value < 0.05). Green indicates upregulation, red indicates downregulation, and black indicates no change in expression. The columns represent individual samples. **B.** Functional pathway enrichment analysis of differentially regulated genes that encode for mitochondrial-localized proteins in iPSC-derived neurons derived from AS patients and healthy controls. The y-axis shows the significantly enriched pathway (FDR < 0.05). The x-axis indicates –ln *p-*value of enrichment of the given process.

The second transcriptomics dataset of AS models that we utilized for examining the expression profiles of genes localized to mitochondria was an RNA sequencing dataset generated from iPSC-derived neurons from AS patients and healthy donor controls [57] (see Section 4). In this dataset, we identified 27 differentially expressed genes (*p-*value < 0.05) (Figure 7A, Table S15). Performing pathway enrichment analysis, we found that the most enriched pathways are directly associated with mitochondrial morphogenesis and translation of mitochondrial coded genes (Figure 7B).

## 3. Discussion

AS is a neurodevelopmental disorder caused by the lack of expression of the UBE3A protein, usually due to a deficiency of the maternal copy of the *UBE3A* gene. Although the genetic etiology of AS is clear, the exact cellular mechanisms underlying the AS pathophysiology remain to be discovered. Over the last two decades, a substantial body of literature has demonstrated the involvement of mitochondrial dysfunction in neurodevelopmental disorders, such as autism [50], Rett syndrome [46,69], and Down syndrome [51]. In addition, recent studies performed by us and others examining the mitochondrial morphology and metabolic-related pathways in AS mouse models revealed alterations in the mitochondrial structure and activity [31,33,35]. Therefore, in this study, we aimed to elucidate the transcriptomic alterations that arise from the deletion of *Ube3a* with an emphasis on the mitochondrial-related pathways.

In the current study, we utilized mouse embryonic fibroblasts (MEFs) as a screening system and an exploratory tool to identify pathways that are dysregulated by *Ube3a* deletion. In the past, MEFs have been shown to be a powerful discovery tool for the identification of molecular pathways relevant to neurodegenerative disorders [70]. Moreover, MEFs lacking the expression of Ube3a were proven to be suitable for studying cellular response to stress [16]. Although *Ube3a* may entail multiple possible transcripts, only three transcripts were shown to be active and entail differential localization and possibly differential roles [9,11,12]. In the herein *Ube3a^−/−^* MEFs, all transcripts were deleted and no Ube3a protein was expressed [19]. 

We first compared the transcriptomes of *Ube3a*^+/+^ and *Ube3a*^−/−^ MEFs. We found 186 genes that were differentially regulated in the *Ube3a* knockdown MEFs (Figure 1A) compared to controls. This analysis revealed several pathways related to mitochondrial functioning such as FoxO signaling and glutathione metabolism (Figure 1B, Supplementary Figure S1). These pathways are an essential mechanism of cellular defense against oxidative stress. FoxO factors reduce cellular ROS production by activating the mitochondrial antioxidant enzymes and remodeling the damaged mitochondria [61], while the glutathione system inhibits the damaging effect of ROS by reducing molecules such as hydrogen peroxide (H_2_O_2_) to water (H_2_O) [67]. Our finding of FoxO and glutathione dysregulation may indicate potential altered ROS levels in *Ube3a*^−/−^ cells coinciding with previous studies that demonstrated elevated oxidative stress levels in CA1 hippocampal neurons of AS model mice [31,33,35]. Interestingly, it has been demonstrated that reducing the oxidative stress by either mito-Q or idebenone Q10 improves the synaptic plasticity and memory impairments of AS mice [33,34].

The mitochondria are the primary sites of ROS production. Under normal physiological conditions, 0.2-2% of the electrons in the electron transport chain (ETC) leak out and interact with oxygen producing ROS like superoxide or hydrogen peroxide, which if not eliminated properly may result in mitochondrial damage and cell death [71]. Alteration of ROS production may arise from multiple metabolic pathways. To further investigate the potential mitochondrial alteration in *Ube3a*^−/−^ MEFs, we analyzed the transcriptome differences in light of genes that encode for mitochondrial-localized proteins which are represented in the MitoCarta2 database [55]. The MitoCarta2 provides a framework for systematic study of mitochondrial function and physiology at transcriptional level. By utilizing the MitoCarta2 database we identified mitochondrial-localized genes in the gene expression matrix of *Ube3a^+/+^* and *Ube3a^-/-^* MEFs. Performing principal component analysis based on this gene expression matrix we found that these mitochondrial-localized genes clearly differentiate between the *Ube3a*^+/+^ and *Ube3a*^−/−^ MEFs (Figure 1C–D).

This baseline quiescent state of cells might not suffice to discover a more comprehensive set of genes that are related to mitochondrial dysfunction, which was associated with AS. Hence, to further study the mitochondrial-related transcriptome differences, it is essential to induce perturbation that will stimulate the mitochondrial-related pathways. For that, we stimulated the MEFs with 25ng/mL of TNFα for 16 h. Tumor necrosis factor-α (TNFα) induces diverse signaling pathways that result in altering the spatial distribution of mitochondria, mitochondrial membrane potential (MMP), cellular respiration, and ROS production [39,40,41]. Moreover, previous studies have provided evidence that ROS produced by TNFα stimulation originate from the mitochondria [72]. To examine whether TNFα treatment induces different cellular transcriptional response associated with mitochondrial activity, we analyzed the expression of genes in response to TNFα stimulation in both *Ube3a*^+/+^ and *Ube3a*^−/−^ MEFs. We found that TNFα induced substantial changes in both *Ube3a*^+/+^ and *Ube3a*^−/−^ MEFs, and some of these differentially expressed genes were common to both *Ube3a*^+/+^ and *Ube3a*^−/−^ MEFs (Figure 2). Functional enrichment analysis performed on these genes identified multiple pathways including TNFα signaling and mitochondrial- and ROS-related pathways (Figure 3), which confirmed that TNFα treatment is a suitable approach for studying mitochondrially related stress.

Next, in order to evaluate the implications of Ube3a deletion on the response to TNFα regarding mitochondrial function and ROS production, we utilized 2X2 factor regression analysis identifying genes differentially affected by both genotype and TNFα treatment factors. Thus, we identified 275 genes that revealed 17 enriched pathways, of which five are directly linked to response to ROS production (Figure 4A). The HIF-1 signaling, regulation of apoptosis, and oxidoreductase pathways were significantly affected by both genotype and TNFα treatment factors (Figure 4B,C). The glutathione and FoxO pathways, which were found to be activated by TNFα treatment in both Ube3a^+/+^ and Ube3a^−/−^ MEFs, were also affected by genotype factor alone as shown in the previous analysis (Figure 1B). The HIF-1 signaling pathway has previously been found to contribute to mitochondrial activity and ROS formation [73]. Interestingly, the *Hif1an* gene, which negatively regulates the transcriptional activity of HIF-1α, has been suggested as a candidate substrate of UBE3A [74]. Furthermore, elevated levels of ROS lead to enhanced activity of mitochondria apoptotic pathways in mediating the extrinsic cell-death pathway [75]. This coincides with our findings of 12 dysregulated apoptosis-related genes that are associated with mitochondrial function (Figure 4C, Table S10). The differential expression of the FoxO, HIF-1, and glutathione pathways may indicate altered response to ROS, while the expression differences in the oxidoreductase pathway may represent the capacity of the mitochondria to catalyze the transfer of electrons that leads to differences in ROS production [68]. Concurring with the aforementioned findings, additional analysis of ROS-related genes as extracted from the GO term ‘response to ROS’ pathway also shows that many of these genes have significant genotype and TNFα effects (Figure 5A). This further emphasizes the role of UBE3A in ROS metabolism.

While all of the above clearly indicate some relation between *Ube3a* deletion and TNFα-induced ROS production, it is still unclear which of these differences are the result of a homeostatic response to altered ROS production in *Ube3a* knockout MEFs and which are causing the altered ROS production. Supportive evidence for the latter option can be found in a study that assigned a novel role for UBE3A in controlling cellular response to oxidative stress [76].

Since differences in cellular ROS production are usually associated with either metabolic or functional aberrations in the mitochondria, we also examined gene expression profiles incorporated in the MitoCarta2 database [55]. We found 36 genes affected by both factors (genotype and TNFα treatment) that are known to localize to the mitochondria (Figure 5B). This also suggests a dysregulation of mitochondrial activity in the absence of UBE3A.

Since all of the abovementioned analyses were performed based on the in vitro evaluations of the transcriptomes of *Ube3a*^+/+^ and *Ube3a*^−/−^ MEFs, we also tested the relevance of the herein findings for AS etiology. For that, we analyzed previously published transcriptome datasets of mouse and human AS models using bioinformatics and machine learning approaches. Analysis of our own transcriptome dataset generated from hippocampi of AS model mice and their WT littermates [56] yielded four mitochondrial-localized genes that were differentially expressed (Figure 6A). However, sometimes the differential expression of a single gene does not entail statistical significance on its own, but as an aggregate of genes, small incremental changes in expression make an overall difference. Therefore, we also utilized the multirun RF approach that identifies genes which, taken together as a group, differentiate between the two genotypes. This approach identified a set of 50 mitochondrial-localized genes that, based on their expression, can differentiate between the WT and the AS mice (Figure 6B,C). In addition, we analyzed a publicly available transcriptome dataset generated from iPSC-derived neurons taken from AS patients and healthy donors controls [57]. This analysis revealed 27 mitochondrial-localized genes that were differentially expressed in the iPSCs-derived neurons from AS patients (Figure 7). To further elucidate the role of *Ube3a* in regulating mitochondrial functions, we examined publicly available gene expression profiles derived from brain tissue of autism spectrum disorder patients with dup15q, in which UBE3A is highly expressed, compared to gene expression profiles of healthy donors [59]. Performing functional cluster analysis on the differentially expressed genes (*p-*value < 0.01 as reported by the authors) in dup15q patients compared to healthy controls, the ‘mitochondrion’ gene ontology cluster was highly significant (Figure S3A). Surveying the genes included in the mitochondrion cluster, we found that 68% of these were downregulated (Figure S3B, Table S16). Similarly, genes known to be localized to the mitochondria (MitoCarta2 [55])and that were found to be differentially expressed in dup15q compared to healthy donors were also mostly (70%) downregulated (Figure S3C, Table S17). This finding is especially interesting because our analysis of iPSC-derived neurons from AS patients, where UBE3A is lacking, showed only upregulation of mitochondrial genes (Figure 7). Additional support for our claims of disrupted mitochondrial pathways in AS can be found also in the proteomics data recently published by Wang et al. [58]. We investigated the proteins found as differentially expressed and that are known to be localized to the mitochondria (MitoCarta2 [55]). Similar to the threshold taken by the authors, we set the threshold for difference in expression as the ratio Z score of Heavy (WT)/Light (AS) >2 or <−2. We found that in the absence of UBE3A, the expression pattern of several mitochondrial-related proteins were disrupted in all the investigated brain regions, cerebellum, cortex, and hippocampus. Interestingly, a different pattern of protein expression was observed in each brain region (Figure S4, Table S18). These findings emphasize the need to investigate the unique roles of UBE3A as a regulator of mitochondrial function in distinct brain regions, especially in light of the possible involvement of UBE3A in early developmental stages in AS pathophysiology [19]. All of the above analyses of different AS and 15q duplication models strengthen the link we found between UBE3A expression and mitochondrial functioning.

Taken together, the aforementioned findings indicate that UBE3A is linked to mitochondrial functioning and ROS production, and that either inactivation or overexpression of UBE3A correlate with changes in mitochondrial-related gene and protein expression profiles. This study is one tier in the exploration of the links between mitochondrial functioning and gene expression profiles in AS. It emphasizes the need for further investigations of mitochondrial functioning in AS and suggests focusing on pathways related to mitochondrial regulation. Better understanding of the links between mitochondrial dysregulation and the resultant alterations of aberrant pathways and gene expression has the potential to impact the development of novel therapeutic approaches for other neurodevelopmental disorders beyond AS.

## 4. Methods

### 4.1. MEFs Generation

Mice used were all on a C57BL/6 background. The MEFs from null (*Ube3a*^−/−^) and WT (*Ube3a*^+/+^) 13.5-day-old embryos were generated by breeding *Ube3a*^−/+^ mice [77] (Supplementary Figure S3A). For the MEF isolation, embryos from 13.5-day-pregnant mice were washed with phosphate-buffered saline (PBS). The head and visceral tissues were removed and the remaining bodies were washed in fresh PBS, minced using a pair of scissors. MEFs cells were isolated using the Primary Mouse Embryonic Fibroblast Isolation Kit #88279 (Thermo, Rockford, IL, USA) according to the manufacturer’s instructions. Cells were collected by centrifugation (200 × g for 5 min at 4 °C) and resuspended in fresh DMEM medium with 15% FBS. Then, 1 × 10^6^ cells were cultured on 100 mm dishes at 37 °C with 5% CO2. In this study, we used MEFs within three to five passages to avoid replicative senescence. Housing, handling, and experimental procedures were performed in accordance with the National Institutes of Health guidelines and were reviewed and approved by the University of Haifa animal ethics committee (ethics # 576/18). 

### 4.2. TNFα Treatment

TNFα cat.# 315-01A-50 was purchased from PEPROTECH (Rocky Hill, NJ, USA). TNFα was initially dissolved in PBS 0.1% BSA according to manufacturer’s protocol. Following, TNFα was dissolved in the medium to a final concentration of 25 ng/mL. We replaced the medium of half of the dishes of each genotype TNFα-containing medium and the other half of the dishes with a similar medium without TNFα. We harvested the MEFs for RNA isolation after 16 h of incubation in the mediums.

### 4.3. RNA-Seq Library Preparation

The *Ube3a*^−/−^ and *Ube3a*^+/+^ MEFs that were used for mRNA sequencing were cultured for four passages and either treated or not treated with TNFα as abovementioned. After trypsinization, cells were collected and total RNA was isolated using the RNeasy Lipid Tissue Mini Kit, Cat No: 74804 (QIAGEN, Austin, TX, USA) according to the manufacturer’s instructions. The mRNA was isolated using polyA selection according to the manufacturer’s instructions (kit Cat.# C05010047 CATS-mRNAseq-kit-manual (with polyA selection) v2). The isolated mRNA concentration and quality were determined by Qubit^®^ quantitation assay using Qubit^®^ 2.0 fluorometer (Invitrogen/Life Technologies, Carlsbad, CA, USA) and Agilent 4200 TapeStation System (Agilent Technologies, Santa Clara, CA, USA). Samples were prepared for Illumina sequencing using NEB’s Ultra RNA Library Prep Kit for Illumina (NEB#7530) (BioLabs Inc, MA, USA) according to the manufacturer’s protocol. Libraries were sequenced in the Technion Genome Center (Haifa, Israel) with a 2 × 150 bp PE run on Illumina HiSeq 2500 (San Diego, CA, USA) using a V3 flow cell.

### 4.4. Bioinformatics Analysis

RNA sequencing data was processed by the Tauber Bioinformatics Research Center parallelized pipeline. The raw reads were cleaned from adaptors according to manufacturer’s instructions of the kit Cat.# C05010047 CATS-mRNAseq-kit-manual (with polyA selection) v2. The cleaned reads were aligned to mouse genome assembly GRCM38:mm10 https://www.ncbi.nlm.nih.gov/assembly/GCF_000001635.20/ using HiSat2 algorithm [78] with ‘--rna-strandness FR’ parameter for alignment of reads generated by directed sequencing approach. The expression levels were quantified by HTSeq [79] in FPKM units. Differentially expressed genes were identified by DeSeq2 algorithm using raw read counts [60].

For further analysis, the expression table was quantile normalized with threshold 5 and transformed to natural logarithmic scale. The normalized table was filtered leaving genes with expression levels higher than three FPKM at least in one sample. One TNFα-treated *Ube3a*^−/−^ (sample 10) was discarded from further analysis due to the low number of acceptable quality reads in the sample compared to all other generated samples. Genotyping of samples was reconfirmed by analyzing the alignment of reads on Ube3a exon2 (Supplementary Figure S3B).

### 4.5. Factor Regression Analysis

In order to identify genes differentially affected by TNFα treatment in *Ube3a*^+/+^ and *Ube3a*^−/−^ MEFs, we utilized a 2 × 2 factorial regression model with finite orthogonal Chebyshev polynomials for estimating main effects (genotype and TNFα treatment) and interaction effect [80]. Significance for main effects was determined at Benjamini–Hochberg adjusted *p-*value < 0.01.

DAVID [81] software was used for functional annotation of genes. We used KEGG [82], GO [83], and MGI [84] to assign genes to pathways and GO functional clusters. To identify mitochondria-related genes, we utilized the MitoCarta2 database [55]. The Heatmapper [85] tool was used to visualize the heat maps for found pathways and functional clusters. We utilized complete linkage clustering method and Pearson distance measure for clustering the genes in each pathway. For each gene, the color scheme represents values above (green) and below (red) the mean expression.

### 4.6. Random Forest Analysis and LDA

Random Forest (RF) is a classification algorithm that has been successfully applied in several recent RNA sequencing studies [86,87]. RF identifies genes that, based on their expression, are possible to classify between the known groups of samples (*Ube3a*^+/+^ and *Ube3a*^−/−^ MEFs). We added an iterative procedure to the standard random Forest R package [88] to identify genes most frequently chosen for the prediction model. We used the 1000-tree RF iterative procedure with 1000 iterations. Linear Discriminant Analysis (LDA) was performed with R MASS package [89] to further validate the prediction power of the chosen genes.

### 4.7. Publicly Available RNA Sequencing Data

In order to validate our results from the *Ube3a*^+/+^ and *Ube3a*^−/−^ MEFs dataset, we utilized our previously published RNA sequencing data generated from hippocampi of AS model mice and their WT control littermates (GEO project accession: PRJNA48422). The second dataset was an RNA sequencing dataset generated from patient-derived iPSC-derived neurons and compared to iPSC-derived neurons derived from healthy donors [57] (SRA accession number: SRP044749). The raw reads were cleaned from adaptors and PCR duplicates using Trimmomatic algorithm [90]. The cleaned reads were aligned either to mouse genome assembly GRCM38:mm10 https://www.ncbi.nlm.nih.gov/assembly/GCF_000001635.20/ in case of mouse derived data or to human genome assembly GRCh38 https://www.ncbi.nlm.nih.gov/assembly/GCF_000001405.26/ when the data was generated from human cell cultures using Bowtie2 algorithm [91]. The expression levels were quantified by RSEM [92] in FPKM units. Each independent expression table was transformed to natural logarithmic scale and quantile normalized with threshold five. The normalized tables were filtered leaving genes with expression levels higher than three FPKM at least in one sample.

Further validation of our results was by investigating the opposite condition of dup15q, where UBE3A is overexpressed. For that, we utilized DAVID functional annotation software [81] for functional clustering of differentially expressed genes (*p-*value < 0.01) in postmortem brains of dup15q patients compared to healthy controls. This gene expression table of dup15q patients was obtained from the supplementary information of a publicly available mRNA-seq dataset [59].

### 4.8. Data Availability

Fastq files of RNA-seq data from treated and untreated *Ube3a*^−/−^ and *Ube3a*^+/+^ MEFs are available in GEO (PRJNA634721).

## Supplementary Materials

Supplementary materials can be found at https://www.mdpi.com/1422-0067/21/11/4156/s1.

## Abbreviations

AS	Angelman Syndrome
ROS	Reactive Oxygen Species
TNFα	Tumor Necrosis Factor α
PCA	Principal Component Analysis
LDA	Linear Discriminant Analysis
RF	Random Forest
