# Supplementary material for: Bioinformatics Analyses of the Transcriptome Reveal Ube3a-Dependent Effects on Mitochondrial-Related Pathways
Source: Int J Mol Sci. 2020 Jun 10;21(11):4156. doi: 10.3390/ijms21114156 (PMC7312912; doi:10.3390/ijms21114156)
Supplement: Supplementary file 1 [file ijms-21-04156-s001.zip › ijms-809633-suppl/ijms-809633-supplementary-english done.docx]

**Supplementary Data**


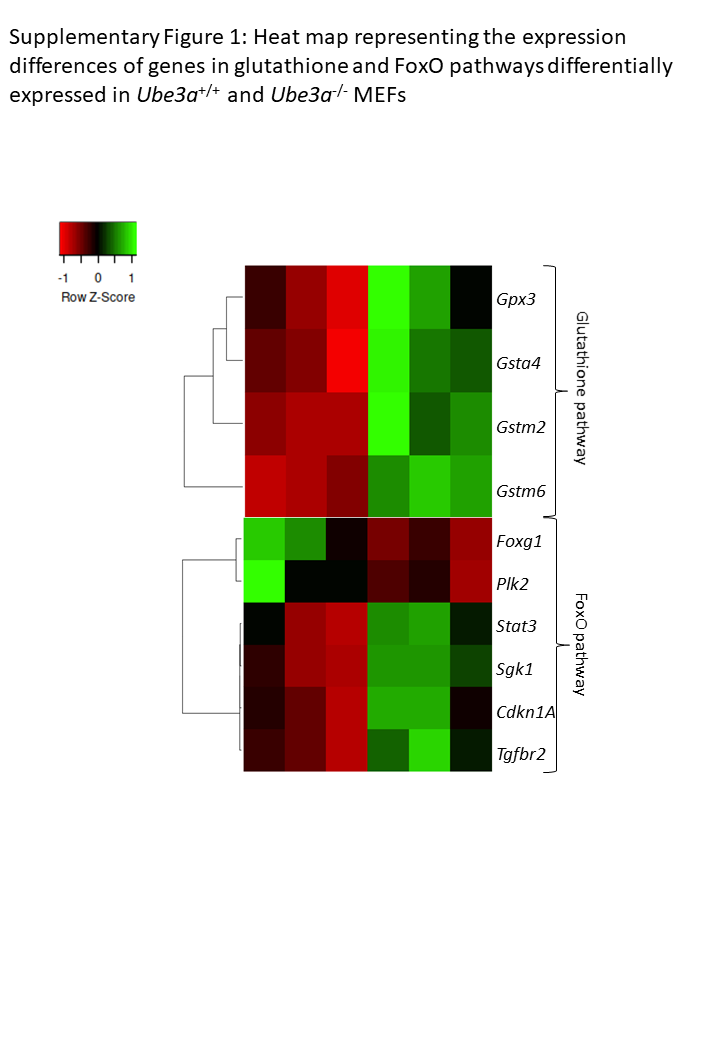


**Figure 1.** Heat map showing different expression pattern of differentially expressed genes in *Ube3a^+/+^* and *Ube3a^-/-^* MEFs in glutathione (4 genes) and FoxO (6 genes) pathways. The heat map indicates upregulation (green), downregulation (red), and mean gene expression (black). The columns represent individual samples.


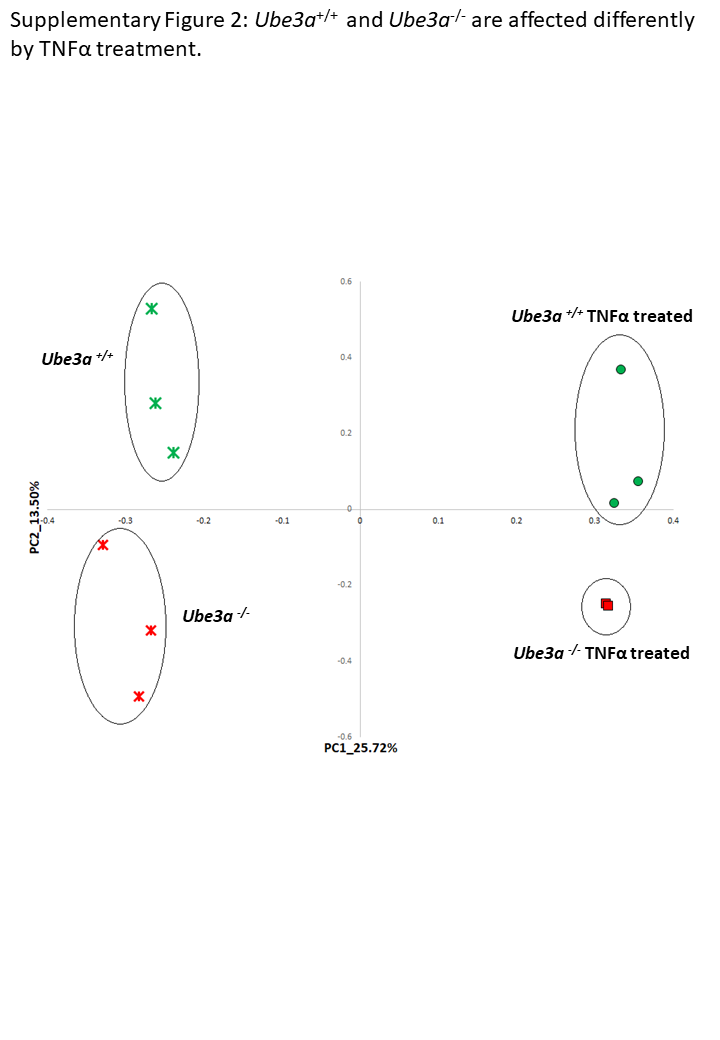


**Figure S2.** Principal Component Analysis (PCA) of genes expressed in *Ube3a^+/+^* and *Ube3a^-/-^* MEFs with vehicle or TNFα treatment revealed that two PCs (PC1 and PC2) reliably separate the samples into four distinct groups. PC1 component separates the vehicle-treated samples from samples treated with TNFα (vehicle-treated samples are denoted by asterisks and TNFα-treated are denoted by circles). Component PC2 separates the samples by genotype (green for *Ube3a^+/+^* and red for *Ube3a^-/-^*).


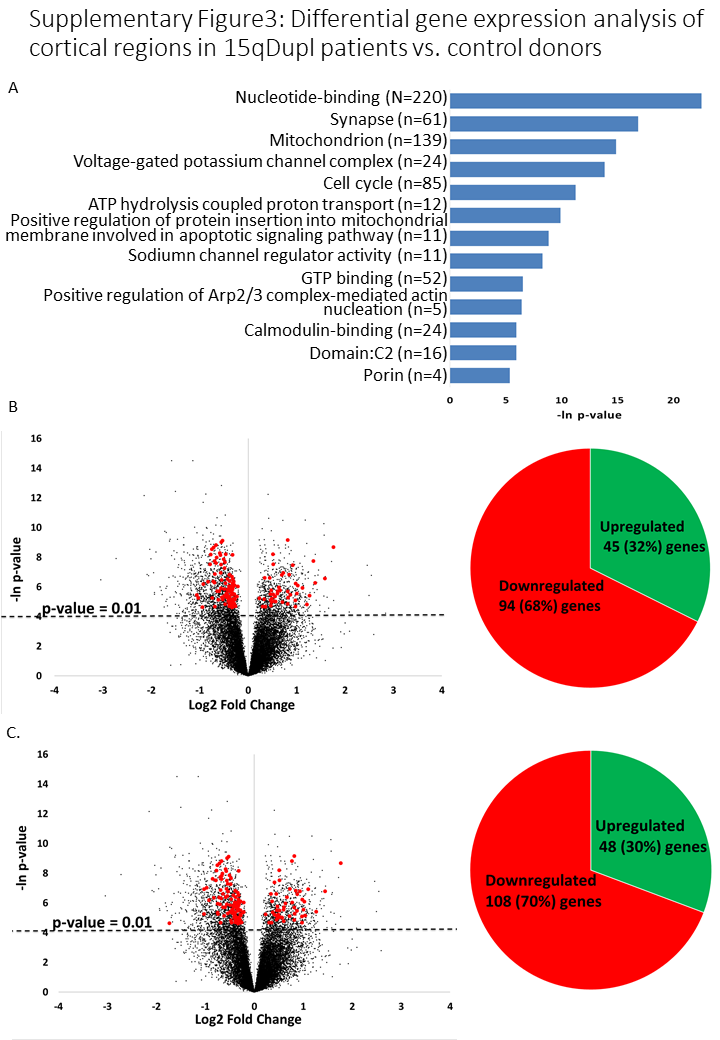


**Figure 3.** Differential gene expression analysis of cortical regions from dup15q patients vs. control donors (<https://doi.org/10.1038/nature20612>). (**A**) Functional pathway enrichment analysis of 1769 significant differentially affected genes in dup15q patients compared to healthy control donors. The threshold of significance of differential expression was set at p-value <0.01 which was determined by the authors of original paper. (**B**) Volcano plot showing the distribution of the gene expression fold changes (x-axis) and p-values (y-axis) in samples from dup15q and healthy control donors. Genes belonging to functional cluster ‘mitochondrion’ and with p-value <0.01 are indicated in red. Pie chart indicating the up- and downregulated genes belonging to ‘mitochondrion’ cluster. Upregulated genes are indicated by the green color. Downregulated genes are indicated in red color. (**C**) Volcano plot showing the distribution of the gene expression fold changes (x-axis) and p-values (y-axis) in samples from dup15q and healthy control donors. Genes known to localize to the mitochondria (MitoCarta2.0) and with p-value <0.01 are indicated in red. Pie chart indicating the up- and downregulated genes known to localize to the mitochondria (MitoCarta2.0). Upregulated genes are indicated by the green color. Downregulated genes are indicated in red color.


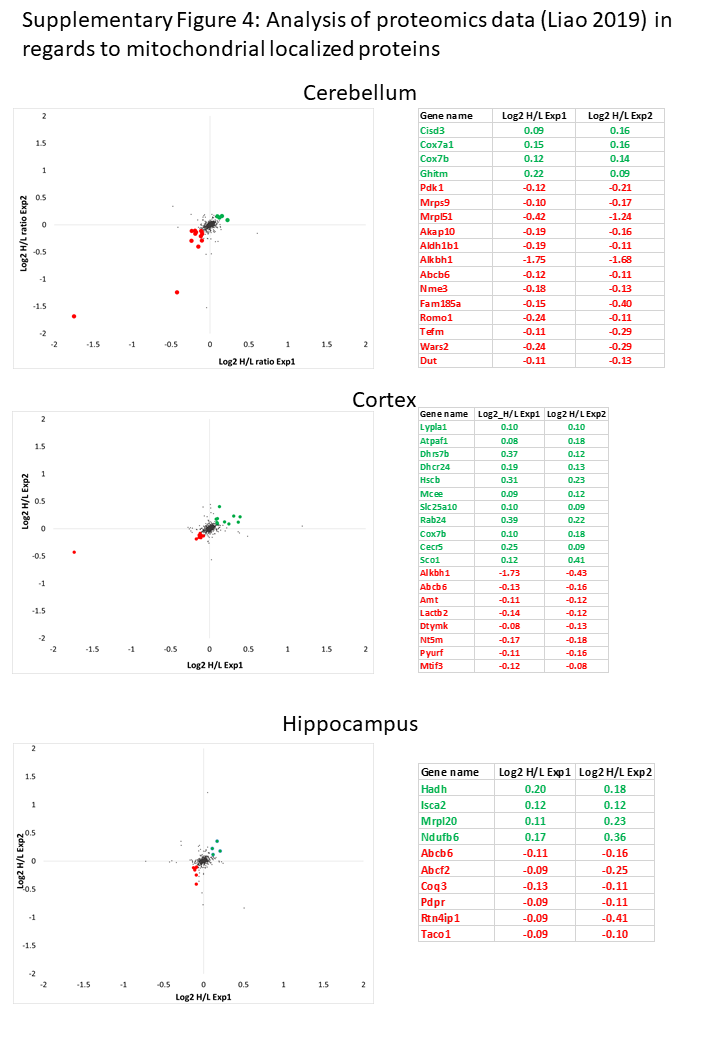


**Figure 4.** Analysis of proteomics data from Wang et al. (https://doi.org/10.1016/j.nbd.2019.104585) in regard to mitochondrial localized proteins (MitoCarta2.0) in three brain regions (cerebellum, cortex, and hippocampus). Correlation plots of two biological repeats showing the up- (indicated by green color) or downregulated (indicated by red color) proteins. Table of mitochondrial localized and significantly up- (green) or downregulated (red) proteins.

**Supplementary Tables:**

Table S1: DeSeq2 differentially expressed genes in *Ube3a^-/-^* MEFs compared to *Ube3a^+/+^* MEFs. 121 genes were upregulated and 65 were downregulated in *Ube3a^-/-^* cells compared to *Ube3a^+/+^* MEFs. The expression values are reported in read counts.

Table S2: Expression values of MitoCarta2 genes expressed in *Ube3a^-/-^* and *Ube3a^+/+^* MEFs. 1080 genes are expressed in this transcriptome dataset. The expression profiles are reported after quantile normalization in FPKM values in natural logarithmic scale.

Table S3: Nine MitoCarta2 genes found by utilizing DeSeq2 algorithm as significantly altered in *Ube3a^-/-^* MEFs compared to *Ube3a^+/+^* MEFs. The expression values are reported in read counts.

Table S4: DeSeq2 differentially expressed genes in *Ube3a*^+/+^ following treatment with TNFα compared to untreated *Ube3a*^+/+^ MEFs. 914 genes were significantly upregulated and 1304 genes were significantly downregulated in *Ube3a*^+/+^ following treatment with TNFα compared to untreated *Ube3a*^+/+^ MEFs. The expression values are reported in read counts.

Table S5: DeSeq2 differentially expressed genes in *Ube3a*^-/-^ following treatment with TNFα compared to untreated *Ube3a*^-/-^ MEFs. 789 genes were upregulated and 1228 were downregulated in TNFα treated *Ube3a*^-/-^ compared to untreated *Ube3a*^-/-^ MEFs. The expression values are reported in read counts.

Table S6: DeSeq2 differentially expressed genes in both *Ube3a*^+/+^ and *Ube3a*^-/-^ cells. 578 genes were significantly upregulated and 930 genes were significantly downregulated when treated with TNFα. The expression values are reported in read counts.

Table S7: DeSeq2 differentially expressed mitochondrial-localized genes (MitoCarta2) showing significant response to TNFα in both *Ube3a*^+/+^ and in *Ube3a*^-/-^ MEFs. The expression profiles are reported after quantile normalization in FPKM values in natural logarithmic scale.

Table S8: 2X2 factor regression analysis yields 275 genes that are significantly affected by the factors of genotype and TNFα treatment in *Ube3a^+/+^* and *Ube3a^-/-^* MEFs (Benjamini–Hochberg adjusted for multiple comparisons p < 0.01). The expression profiles are reported after quantile normalization in FPKM values in natural logarithmic scale.

Table S9: 2X2 factor regression analysis yields 24 genes that are significantly affected by the factors of genotype and TNFα treatment in *Ube3a^+/+^* and *Ube3a^-/-^* MEFs (Benjamini–Hochberg adjusted for multiple comparisons p < 0.01). These genes are assigned to the GO pathways: ‘Glutathione metabolic process’, ‘FoxO signaling pathway’, ‘HIF-1 signaling pathway’, and ‘Oxidoreductase pathway’. The expression profiles are reported after quantile normalization in FPKM values in natural logarithmic scale.

Table S10: 2X2 factor regression analysis yields 12 genes that are significantly affected by the factors of genotype and TNFα treatment in *Ube3a^+/+^* and *Ube3a^-/-^* MEFs (Benjamini–Hochberg adjusted for multiple comparisons p < 0.01). These genes are assigned to the GO pathway of ‘Positive regulation of apoptosis’. The expression profiles are reported after quantile normalization in FPKM values in natural logarithmic scale.

Table S11: 2X2 factor regression analysis yields 29 genes that are significantly affected by the factors of genotype and TNFα treatment in *Ube3a^+/+^* and *Ube3a^-/-^* MEFs (Benjamini–Hochberg adjusted for multiple comparisons p < 0.01). These genes are assigned to the GO pathway of ‘ROS metabolic process’. The expression profiles are reported after quantile normalization in FPKM values in natural logarithmic scale.

Table S12: 2X2 factor regression analysis yields 36 genes that are significantly affected by the factors of genotype and TNFα treatment in *Ube3a^+/+^* and *Ube3a^-/-^* MEFs (Benjamini–Hochberg adjusted for multiple comparisons p < 0.01). These genes are known to be mitochondrial-localized proteins (taken from MitoCarta2). The expression profiles are reported after quantile normalization in FPKM values in natural logarithmic scale.

Table S13: Four mitochondria-localized genes differentially expressed (p-value <0.05) in AS and WT mouse hippocampi. The expression profiles are reported after quantile normalization in FPKM values in natural logarithmic scale.

Table S14: Multirun random forest (multirun RF) procedure yielded 50 classifier genes known to localize to the mitochondria (taken from MitoCarta2). These 50 genes, as a group, clearly differentiate between AS or WT hippocampi. The expression profiles are reported after quantile normalization in FPKM values in natural logarithmic scale.

Table S15: Analysis of the iPSC-derived neurons from AS patients and healthy donors controls dataset yields 27 differentially expressed genes (p-value <0.05) that are localized to the mitochondria (taken from MitoCarta2). The expression profiles are reported after quantile normalization in FPKM values in natural logarithmic scale.

Table S16: Differentially expressed genes in dup15q patients compared to healthy controls (p-value <0.01 as reported by Parikshak et al. Nature 2016). These genes are assigned to the GO term ‘mitochondrion’.

Table S17: Differentially expressed genes in dup15q patients compared to healthy controls (p-value <0.01 as reported by Parikshak et al. Nature 2016). These genes are known to be localized to the mitochondria (MitoCarta2).

**Table S18.** Differentially expressed proteins known to be localized to the mitochondria (MitoCarta2) as expressed in three brain regions: cerebellum, cortex, and hippocampus (Wang et al. Neurobiology of Disease 2019). The threshold of significance was set similar to the original authors as the ratio Z score of Heavy (WT)/Light (AS) >2 or <-2.

|  | Gene name | Exp1_Ceb_H/L | Exp2_Ceb_H/L | Mean_Ceb_H/L | **Signifi_Ceb_H/L** |
| --- | --- | --- | --- | --- | --- |
| Cerebellum | Cisd3 | 1.2373 | 1.441 | 1.33915 | **1** |
|  | Cox7a1 | 1.4194 | 1.4604 | 1.4399 | **1** |
|  | Cox7b | 1.3178 | 1.3704 | 1.3441 | **1** |
|  | Ghitm | 1.671 | 1.2218 | 1.4464 | **1** |
|  | Pdk1 | 0.75837 | 0.6161 | 0.687235 | **-1** |
|  | Mrps9 | 0.7888 | 0.68139 | 0.735095 | **-1** |
|  | Mrpl51 | 0.37589 | 0.057293 | 0.2165915 | **-1** |
|  | Akap10 | 0.64236 | 0.68693 | 0.664645 | **-1** |
|  | Aldh1b1 | 0.63903 | 0.78463 | 0.71183 | **-1** |
|  | Alkbh1 | 0.017953 | 0.020866 | 0.0194095 | **-1** |
|  | Abcb6 | 0.76709 | 0.77454 | 0.770815 | **-1** |
|  | Nme3 | 0.65816 | 0.73544 | 0.6968 | **-1** |
|  | Fam185a | 0.70529 | 0.39707 | 0.55118 | **-1** |
|  | Romo1 | 0.57827 | 0.7718 | 0.675035 | **-1** |
|  | Tefm | 0.7808 | 0.51273 | 0.646765 | **-1** |
|  | Wars2 | 0.57664 | 0.51041 | 0.543525 | **-1** |
|  | Dut | 0.78241 | 0.74431 | 0.76336 | **-1** |
| cortex | Lypla1 | 1.2573 | 1.2617 | 1.2595 | **1** |
|  | Atpaf1 | 1.2074 | 1.5051 | 1.35625 | **1** |
|  | Dhrs7b | 2.3255 | 1.3174 | 1.82145 | **1** |
|  | Dhcr24 | 1.5533 | 1.3356 | 1.44445 | **1** |
|  | Hscb | 2.0366 | 1.7023 | 1.86945 | **1** |
|  | Mcee | 1.2362 | 1.3123 | 1.27425 | **1** |
|  | Slc25a10 | 1.257 | 1.2281 | 1.24255 | **1** |
|  | Rab24 | 2.4563 | 1.6533 | 2.0548 | **1** |
|  | Cox7b | 1.2534 | 1.5303 | 1.39185 | **1** |
|  | Cecr5 | 1.7643 | 1.2209 | 1.4926 | **1** |
|  | Sco1 | 1.3309 | 2.548 | 1.93945 | **1** |
|  | Alkbh1 | 0.018478 | 0.37541 | 0.196944 | **-1** |
|  | Abcb6 | 0.73364 | 0.69561 | 0.714625 | **-1** |
|  | Amt | 0.77794 | 0.76192 | 0.76993 | **-1** |
|  | Lactb2 | 0.73082 | 0.767 | 0.74891 | **-1** |
|  | Dtymk | 0.83163 | 0.7376 | 0.784615 | **-1** |
|  | Nt5m | 0.67505 | 0.65485 | 0.66495 | **-1** |
|  | Pyurf | 0.77416 | 0.68456 | 0.72936 | **-1** |
|  | Mtif3 | 0.75741 | 0.82247 | 0.78994 | **-1** |
| hippocampus | Hadh | 1.602 | 1.5105 | 1.55625 | **1** |
|  | Isca2 | 1.3067 | 1.3155 | 1.3111 | **1** |
|  | Mrpl20 | 1.2826 | 1.6859 | 1.48425 | **1** |
|  | Ndufb6 | 1.4666 | 2.2723 | 1.86945 | **1** |
|  | Abcb6 | 0.77078 | 0.69463 | 0.732705 | **-1** |
|  | Abcf2 | 0.80925 | 0.56592 | 0.687585 | **-1** |
|  | Coq3 | 0.74372 | 0.76807 | 0.755895 | **-1** |
|  | Pdpr | 0.80525 | 0.77278 | 0.789015 | **-1** |
|  | Rtn4ip1 | 0.80939 | 0.39052 | 0.599955 | **-1** |
|  | Taco1 | 0.81088 | 0.7916 | 0.80124 | **-1** |
