# Supplementary material for: Bioinformatics Analyses of the Transcriptome Reveal Ube3a-Dependent Effects on Mitochondrial-Related Pathways
Source: Int J Mol Sci. 2020 Jun 10;21(11):4156. doi: 10.3390/ijms21114156 (PMC7312912; doi:10.3390/ijms21114156)
Supplement: Supplementary file 1 [file ijms-21-04156-s001.zip › ijms-809633-suppl/Legends to Figures_SuppFigs_SuppTables_310520.docx]

**Legends**

Supplementary Figure 1:

Heat map showing different expression pattern of differentially expressed genes in *Ube3a^+/+^* and *Ube3a^-/-^* MEFs in Glutathione (4 genes) and FoxO (6 genes) pathways. The heat map indicates up-regulation (green), down-regulation (red), and mean gene expression (black). The columns represent individual samples.

Supplementary Figure 2:

Principal Component Analysis (PCA) of genes expressed in *Ube3a^+/+^* and *Ube3a^-/-^* MEFs with vehicle or TNFα treatment revealed that two PCs (PC1 and PC2) reliably separate the samples into four distinct groups. PC1 component separates the vehicle treated samples from samples treated with TNFα (vehicle treated samples are denoted by asterisks and TNFα treated are denoted by circles). Component PC2 separates the samples by genotype (green for *Ube3a^+/+^* and red for *Ube3a^-/-^*).

Supplementary Figure 3:

Differential gene expression analysis of cortical regions from dup15q patients vs. control donors (<https://doi.org/10.1038/nature20612>)

1. Functional pathway enrichment analysis of 1769 significant differentially affected genes in dup15q patients compared to healthy control donors. The threshold of significance of differential expression was set at p-value<0.01 which was determined by the authors of original paper.
2. Volcano plot showing the distribution of the gene expression fold changes (x-axis) and p-values (y-axis) in samples from dup15q and healthy control donors. Genes belonging to functional cluster ‘mitochondrion’ and with p-value < 0.01 are indicated in red. Pie chart indicating the up and down regulated genes belonging to ‘mitochondrion’ cluster. Upregulated genes are indicated by the green color. Downregulated genes are indicated in red color.
3. Volcano plot showing the distribution of the gene expression fold changes (x-axis) and p-values (y-axis) in samples from dup15q and healthy control donors. Genes known to localize to the mitochondria (MitoCarta2.0) and with p-value < 0.01 are indicated in red. Pie chart indicating the up and down regulated genes known to localize to the mitochondria (MitoCarta2.0). Upregulated genes are indicated by the green color. Downregulated genes are indicated in red color.

Supplementary Figure 4:

Analysis of proteomics data from Wang et al (https://doi.org/10.1016/j.nbd.2019.104585) in regards to mitochondrial localized proteins (MitoCarta2.0) in three brain regions (cerebellum, cortex, and hippocampus). Correlation plots of two biological repeats showing the up (indicated by green color) or down (indicated by red color) regulated proteins. Table of mitochondrial localized and significantly up (green) or down (red) regulated proteins.

**Legends to supplementary tables:**

Supplementary Table 1: DeSeq2 differentially expressed genes in *Ube3a^-/-^* MEFs compared to *Ube3a^+/+^* MEFs. 121 genes were upregulated and 65 were downregulated in *Ube3a^-/-^* cells compared to *Ube3a^+/+^* MEFs. The expression values are reported in read counts.

Supplementary Table 2: Expression values of MitoCarta2 genes expressed in *Ube3a^-/-^* and *Ube3a^+/+^* MEFs. 1080 genes are expressed in this transcriptome data set. The expression profiles are reported after quantile normalization in FPKM values in natural logarithmic scale.

Supplementary Table 3: Nine MitoCarta2 genes found by utilizing DeSeq2 algorithm as significantly altered in *Ube3a^-/-^* MEFs compared to *Ube3a^+/+^* MEFs. The expression values are reported in read counts.

Supplementary Table 4: DeSeq2 differentially expressed genes in *Ube3a*^+/+^ following treatment with TNFα compared to untreated *Ube3a*^+/+^ MEFs. 914 genes were significantly upregulated and 1304 genes were significantly downregulated in *Ube3a*^+/+^ following treatment with TNFα compared to untreated *Ube3a*^+/+^ MEFs. The expression values are reported in read counts.

Supplementary Table 5: DeSeq2 differentially expressed genes in *Ube3a*^-/-^ following treatment with TNFα compared to untreated *Ube3a*^-/-^ MEFs. 789 genes were upregulated and 1228 were downregulated in TNFα treated *Ube3a*^-/-^ compared to untreated *Ube3a*^-/-^ MEFs. The expression values are reported in read counts.

Supplementary Table 6: DeSeq2 differentially expressed genes in both *Ube3a*^+/+^ and *Ube3a*^-/-^ cells. 578 genes were significantly upregulated and 930 genes were significantly downregulated when treated with TNFα. The expression values are reported in read counts.

Supplementary Table 7: DeSeq2 differentially expressed mitochondrial-localized genes (MitoCarta2) showing significant response to TNFα in both *Ube3a*^+/+^ and in *Ube3a*^-/-^ MEFs. The expression profiles are reported after quantile normalization in FPKM values in natural logarithmic scale.

Supplementary Table 8: 2X2 factor regression analysis yields 275 genes that are significantly affected by the factors of genotype and TNFα treatment in *Ube3a^+/+^* and *Ube3a^-/-^* MEFs (Benjamini-Hochberg adjusted for multiple comparisons p<0.01). The expression profiles are reported after quantile normalization in FPKM values in natural logarithmic scale.

Supplementary Table 9: 2X2 factor regression analysis yields 24 genes that are significantly affected by the factors of genotype and TNFα treatment in *Ube3a^+/+^* and *Ube3a^-/-^* MEFs (Benjamini-Hochberg adjusted for multiple comparisons p<0.01). These genes are assigned to the GO pathways: ‘Glutathione metabolic process’, ‘FoxO signaling pathway’, ‘HIF-1 signaling pathway’ and ‘Oxidoreductase pathway’. The expression profiles are reported after quantile normalization in FPKM values in natural logarithmic scale.

Supplementary Table 10: 2X2 factor regression analysis yields 12 genes that are significantly affected by the factors of genotype and TNFα treatment in *Ube3a^+/+^* and *Ube3a^-/-^* MEFs (Benjamini-Hochberg adjusted for multiple comparisons p<0.01). These genes are assigned to the GO pathway of ‘Positive regulation of apoptosis’. The expression profiles are reported after quantile normalization in FPKM values in natural logarithmic scale.

Supplementary Table 11: 2X2 factor regression analysis yields 29 genes that are significantly affected by the factors of genotype and TNFα treatment in *Ube3a^+/+^* and *Ube3a^-/-^* MEFs (Benjamini-Hochberg adjusted for multiple comparisons p<0.01). These genes are assigned to the GO pathway of ‘ROS metabolic process’. The expression profiles are reported after quantile normalization in FPKM values in natural logarithmic scale.

Supplementary Table 12: 2X2 factor regression analysis yields 36 genes that are significantly affected by the factors of genotype and TNFα treatment in *Ube3a^+/+^* and *Ube3a^-/-^* MEFs (Benjamini-Hochberg adjusted for multiple comparisons p<0.01). These genes are known to be mitochondrial-localized proteins (taken from MitoCarta2). The expression profiles are reported after quantile normalization in FPKM values in natural logarithmic scale.

Supplementary Table 13: Four mitochondria-localized genes differentially expressed (p-value<0.05) in AS and WT mouse hippocampi. The expression profiles are reported after quantile normalization in FPKM values in natural logarithmic scale.

Supplementary Table 14: Multi-run random forest (multi-run RF) procedure yielded 50 classifier genes known to localize to the mitochondria (taken from MitoCarta2). These 50 genes, as a group, clearly differentiate between AS or WT hippocampi. The expression profiles are reported after quantile normalization in FPKM values in natural logarithmic scale.

Supplementary Table 15: Analysis of the iPSC-derived neurons from AS patients and healthy donors controls dataset yields 27 differentially expressed genes (p-value<0.05) that are localized to the mitochondria (taken from MitoCarta2). The expression profiles are reported after quantile normalization in FPKM values in natural logarithmic scale.

Supplementary Table 16: Differentially expressed genes in dup15q patients compared to healthy controls (p-value<0.01 as reported by Parikshak et al. Nature 2016). These genes are assigned to the GO term ‘mitochondrion’.

Supplementary Table 17: Differentially expressed genes in dup15q patients compared to healthy controls (p-value<0.01 as reported by Parikshak et al. Nature 2016). These genes are known to be localized to the mitochondria (MitoCarta2).

Supplementary Table 18: Differentially expressed proteins known to be localized to the mitochondria (MitoCarta2) as expressed in three brain regions: cerebellum, cortex, and hippocampus (Wang et al. Neurobiology of Disease 2019). The threshold of significance was set similar to the original authors as the ratio Z score of Heavy (WT)/ Light (AS) >2 or <-2.
