# Supplementary material for: Bioinformatics Analyses of the Transcriptome Reveal Ube3a-Dependent Effects on Mitochondrial-Related Pathways
Source: Int J Mol Sci. 2020 Jun 10;21(11):4156. doi: 10.3390/ijms21114156 (PMC7312912; doi:10.3390/ijms21114156)
Supplement: Supplementary file 1 [file ijms-21-04156-s001.zip › ijms-809633-suppl/SupplementaryTable18 Proteomics data AS vs control three brain regions.docx]

|  | Gene name | Exp1_Ceb_H/L | Exp2_Ceb_H/L | Mean_Ceb_H/L | **Signifi_Ceb_H/L** |
| --- | --- | --- | --- | --- | --- |
| Cerebellum | Cisd3 | 1.2373 | 1.441 | 1.33915 | **1** |
|  | Cox7a1 | 1.4194 | 1.4604 | 1.4399 | **1** |
|  | Cox7b | 1.3178 | 1.3704 | 1.3441 | **1** |
|  | Ghitm | 1.671 | 1.2218 | 1.4464 | **1** |
|  | Pdk1 | 0.75837 | 0.6161 | 0.687235 | **-1** |
|  | Mrps9 | 0.7888 | 0.68139 | 0.735095 | **-1** |
|  | Mrpl51 | 0.37589 | 0.057293 | 0.2165915 | **-1** |
|  | Akap10 | 0.64236 | 0.68693 | 0.664645 | **-1** |
|  | Aldh1b1 | 0.63903 | 0.78463 | 0.71183 | **-1** |
|  | Alkbh1 | 0.017953 | 0.020866 | 0.0194095 | **-1** |
|  | Abcb6 | 0.76709 | 0.77454 | 0.770815 | **-1** |
|  | Nme3 | 0.65816 | 0.73544 | 0.6968 | **-1** |
|  | Fam185a | 0.70529 | 0.39707 | 0.55118 | **-1** |
|  | Romo1 | 0.57827 | 0.7718 | 0.675035 | **-1** |
|  | Tefm | 0.7808 | 0.51273 | 0.646765 | **-1** |
|  | Wars2 | 0.57664 | 0.51041 | 0.543525 | **-1** |
|  | Dut | 0.78241 | 0.74431 | 0.76336 | **-1** |
| cortex | Lypla1 | 1.2573 | 1.2617 | 1.2595 | **1** |
|  | Atpaf1 | 1.2074 | 1.5051 | 1.35625 | **1** |
|  | Dhrs7b | 2.3255 | 1.3174 | 1.82145 | **1** |
|  | Dhcr24 | 1.5533 | 1.3356 | 1.44445 | **1** |
|  | Hscb | 2.0366 | 1.7023 | 1.86945 | **1** |
|  | Mcee | 1.2362 | 1.3123 | 1.27425 | **1** |
|  | Slc25a10 | 1.257 | 1.2281 | 1.24255 | **1** |
|  | Rab24 | 2.4563 | 1.6533 | 2.0548 | **1** |
|  | Cox7b | 1.2534 | 1.5303 | 1.39185 | **1** |
|  | Cecr5 | 1.7643 | 1.2209 | 1.4926 | **1** |
|  | Sco1 | 1.3309 | 2.548 | 1.93945 | **1** |
|  | Alkbh1 | 0.018478 | 0.37541 | 0.196944 | **-1** |
|  | Abcb6 | 0.73364 | 0.69561 | 0.714625 | **-1** |
|  | Amt | 0.77794 | 0.76192 | 0.76993 | **-1** |
|  | Lactb2 | 0.73082 | 0.767 | 0.74891 | **-1** |
|  | Dtymk | 0.83163 | 0.7376 | 0.784615 | **-1** |
|  | Nt5m | 0.67505 | 0.65485 | 0.66495 | **-1** |
|  | Pyurf | 0.77416 | 0.68456 | 0.72936 | **-1** |
|  | Mtif3 | 0.75741 | 0.82247 | 0.78994 | **-1** |
| hippocampus | Hadh | 1.602 | 1.5105 | 1.55625 | **1** |
|  | Isca2 | 1.3067 | 1.3155 | 1.3111 | **1** |
|  | Mrpl20 | 1.2826 | 1.6859 | 1.48425 | **1** |
|  | Ndufb6 | 1.4666 | 2.2723 | 1.86945 | **1** |
|  | Abcb6 | 0.77078 | 0.69463 | 0.732705 | **-1** |
|  | Abcf2 | 0.80925 | 0.56592 | 0.687585 | **-1** |
|  | Coq3 | 0.74372 | 0.76807 | 0.755895 | **-1** |
|  | Pdpr | 0.80525 | 0.77278 | 0.789015 | **-1** |
|  | Rtn4ip1 | 0.80939 | 0.39052 | 0.599955 | **-1** |
|  | Taco1 | 0.81088 | 0.7916 | 0.80124 | **-1** |
