# Supplementary figures and images for: Bioinformatics Analyses of the Transcriptome Reveal Ube3a-Dependent Effects on Mitochondrial-Related Pathways
Source: Int J Mol Sci. 2020 Jun 10;21(11):4156. doi: 10.3390/ijms21114156 (PMC7312912; doi:10.3390/ijms21114156)

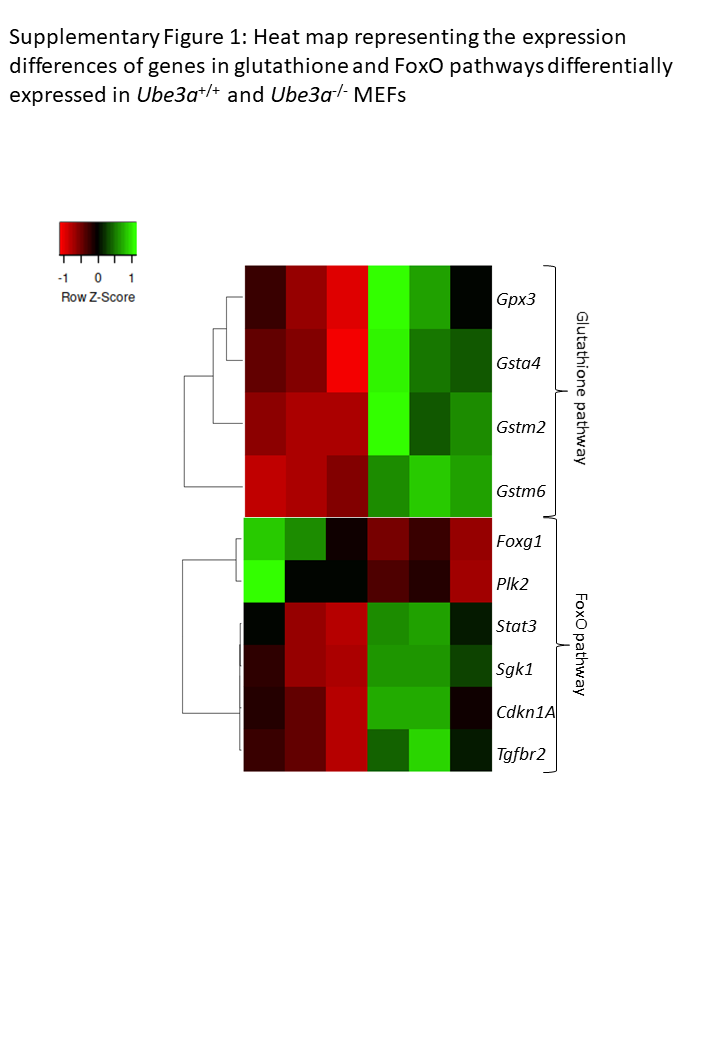

Supplement: Supplementary file 1 [file ijms-21-04156-s001.zip › ijms-809633-suppl/SuppFig-S1.TIF]

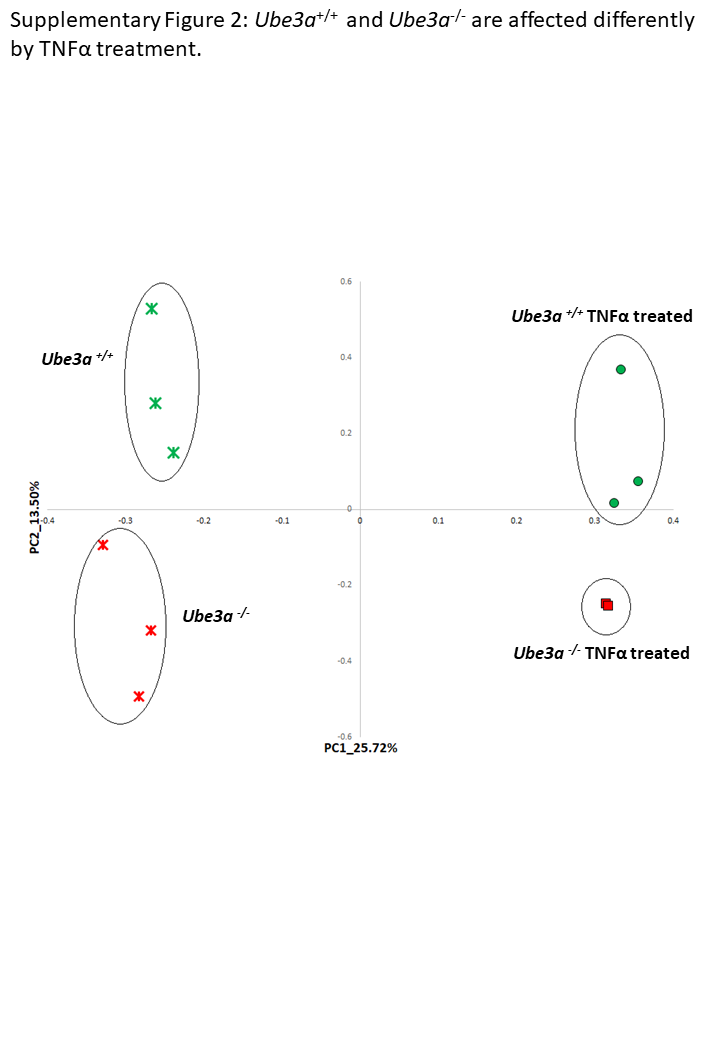

Supplement: Supplementary file 1 [file ijms-21-04156-s001.zip › ijms-809633-suppl/SuppFig-S2.TIF]

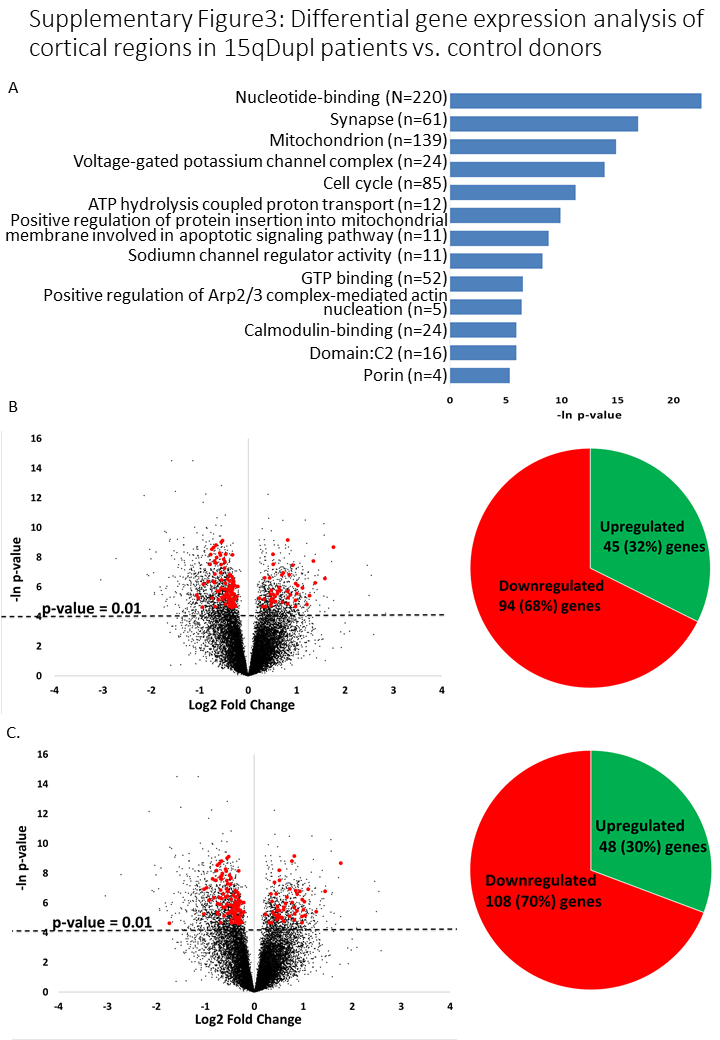

Supplement: Supplementary file 1 [file ijms-21-04156-s001.zip › ijms-809633-suppl/SuppFig-S3.TIF]

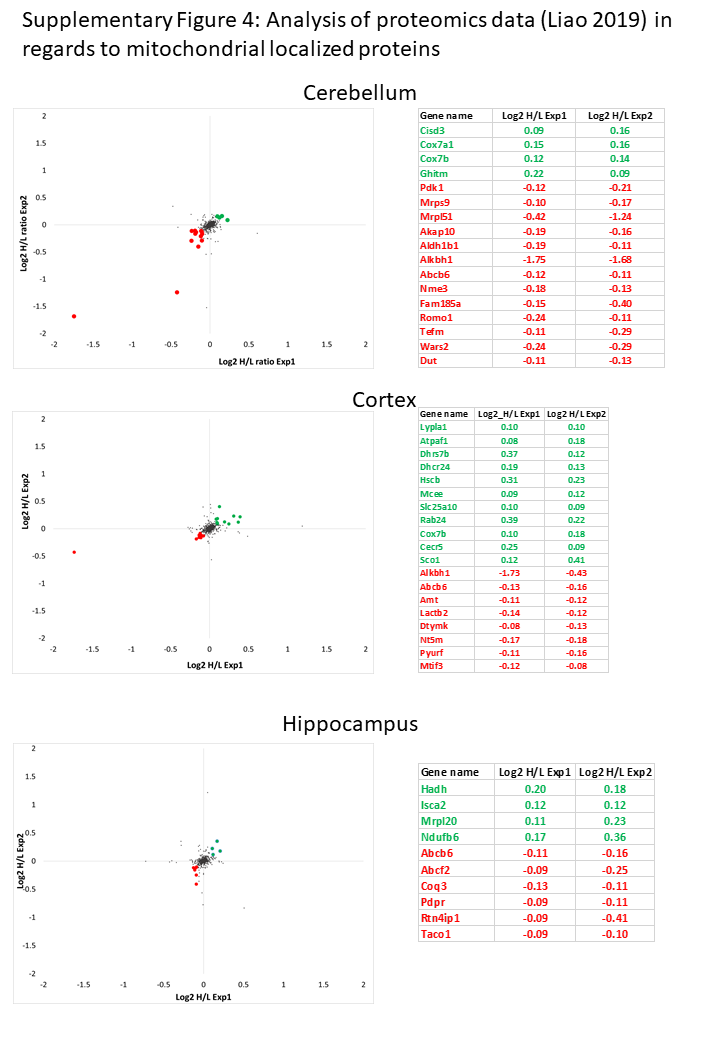

Supplement: Supplementary file 1 [file ijms-21-04156-s001.zip › ijms-809633-suppl/SuppFIg-S4.TIF]
